# Granulomas as the Most Useful Histopathological Feature in Distinguishing between Crohn's Disease and Intestinal Tuberculosis in Endoscopic Biopsy Specimens

**DOI:** 10.1097/MD.0000000000002157

**Published:** 2015-12-11

**Authors:** Ziyin Ye, Yuan Lin, Qinghua Cao, Yao He, Ling Xue

**Affiliations:** From the Department of Pathology, The First Affiliated Hospital, Sun Yat-sen University, Guangzhou, China (ZY, YL, QC, LX); and Department of Gastroenterology, The First Affiliated Hospital, Sun Yat-sen University, Guangzhou, China (YH).

## Abstract

The incidence of Crohn's disease (CD) is increasing in Chinese populations in whom intestinal tuberculosis (ITB) is prevalent.

This study aimed to identify differential diagnostic microscopic and endoscopic characteristics of CD from those of ITB.

Patients with CD (N = 52) and patients with ITB (N = 16) diagnosed between 2010 and 2013 were identified. Specimens obtained via endoscopy were analyzed microscopically by a pathologist. The relationship between endoscopic appearance and histopathological features was analyzed. The χ^2^ test, Fisher's exact probability test, and the Mann-Whitney U test were used.

Granulomas were present in 81.3% of ITB cases and in 67.3% of CD cases (*P* = 0.36). Granulomas in ITB cases were denser than those in CD cases (mean 5.29 ± 4.30 vs. 2.46 ± 3.50 granulomas per 10 low power fields; each low power field = 3.80 mm^2^; *P* = 0.005). Granulomas in ITB cases were larger (mean widest diameter, 508 ± 314 μm; range, 100–1100 μm) than those in CD cases (mean widest diameter, 253 ± 197 μm; range, 50–800 μm). Basal plasmacytosis was more common in CD cases than in ITB cases (77.0% vs. 37.5%, *P* = 0.000). Endoscopy findings such as longitudinal ulcer, aphthous ulcer, and cobblestone appearance were only seen in CD cases (34.6%, 21.2%, and 23.1%, respectively). Granulomas were detected in the majority of cases with longitudinal ulcers (88.9%). Basal plasmacytosis was exclusively detected in cases with longitudinal ulcer and a cobblestone appearance.

Characteristics of granulomas maybe the most important distinguishing features between CD and ITB. However, the histopathological characteristics of both diseases may overlap on endoscopic biopsy specimens. An accurate diagnosis should be made that considers clinical, endoscopic features, and pathologic findings.

## INTRODUCTION

Making the distinction between intestinal tuberculosis (ITB) and Crohn's disease (CD) can be a major diagnostic challenge because both are chronic granulomatous disorders with similar clinical presentations and histopathological features. Although the diagnostic features distinguishing these two diseases have been well described, they were based on studies of surgically resected specimens.^1–4^ However, endoscopic biopsy specimens have become a major diagnostic material. In most cases, a pathological diagnosis is made on endoscopically obtained mucosal biopsies. The most distinctive features, eg. caseation and acid-fast bacilli for ITB as well as fissuring ulcers and transmural inflammation for CD, are usually not present in endoscopically obtained mucosal biopsies.

Although some new techniques such as immunohistochemical (IHC) staining, *Mycobacterium tuberculosis* polymerase chain reaction (PCR), and fluorescence in situ hybridization have recently been introduced to distinguish the two diseases, studies on their sensitivity and specificity are conflicting and their diagnostic utility is uncertain.^5–8^ As a result, histopathological examination still plays an important role in distinguishing between ITB and CD, especially when clinical and endoscopic features are contradictory.

Endoscopy examination of mucosal biopsies is the most common method of diagnosing ITB and CD. Characteristic endoscopic features of ITB and CD have been well described.^9–11^ ITB is characterized by transverse ulcers, nodularity, and hypertrophic lesions resembling masses, while CD is characterized by aphthous or longitudinal, deep, fissuring ulcers and a cobblestone appearance. However, the relationship between endoscopic changes and histopathological features has not been studied.

This study was designed to compare the pathological features of ITB and CD in mucosal biopsies and compare endoscopic changes and histopathological features to set up a morphologic panel to differentiate CD from ITB.

## MATERIALS AND METHODS

### Patients

In this retrospective study, the clinical database of the Department of Gastroenterology, the First Affiliate Hospital of Sun Yat-Sen University, was reviewed to identify patients with CD and ITB diagnosed between 2010 and 2013.

### Diagnostic Criteria

The diagnosis of ITB or CD was confirmed considering clinical, endoscopic, radiological, and histological features, as well as antituberculosis treatment response. The diagnosis of ITB was based on at least one of the following criteria: (a) detection of caseating granuloma on histopathology; (b) positive acid-fast staining for bacilli; (c) *M. tuberculosis* developed on tissue culture; or (d) symptoms relief and endoscopy healing after six months of antituberculosis therapy without recurrence. In addition, patients with concurrent active extraintestinal tuberculosis were considered to be ITB. The diagnosis of CD was considered at least two of the following criteria were met: (a) manifestations of abdominal pain, malaise, weight loss, diarrhea, and/or rectal bleeding; (b) endoscopic lesions including skipping areas, mucosal cobblestoning, linear ulceration, or perianal disease; (c) stricture, fistula, mucosal cobblestoning, or ulceration detected on radiology; (d) laparotomy found bowel wall induration, mesenteric lymphadenopathy, or “creeping fat”; and (e) segmental and transmural inflammation, fissures and/or non-caseous granulomas on histopathological examination of surgically resected specimens. In addition, clinical and endoscopic response to therapy for CD was required to confirm the final diagnosis of CD.^12^ A total of 16 patients with ITB and 52 patients with CD were selected for this study. Biopsies of all cases were taken before definitive treatment was started. This study was approved by the Human Ethics Committee of The First Affiliated Hospital, Sun Yat-sen University.

### Biopsy and Specimens

Colonoscopy was carried out in all patients using an Olympus fiberoptic colonoscope after appropriate preparation. Abnormal areas of the mucosa were noted considering the location and features. An average of six mucosal fragments was obtained from each abnormal site.

The lesions were defined as follows:

Aphthous ulcer: a small, discreet ulcer surrounded by an erythematous halo.

Longitudinal ulcer: an ulcer >5 cm that runs longitudinally along the intestinal tract.

Circumferential ulcer: a linear ulcer in a circumferential arrangement.

Cobblestone appearance: Dense protrusions of mucous membranes of uneven large or small sizes surrounded by longitudinal and small ulcers.

Biopsy specimens were fixed in buffered formaldehyde, embedded in paraffin wax, and serially sectioned at 5 μm and stained with hematoxylin and eosin. An average of six sections was present on each slide.

### Microscopic Analysis

Slides were reviewed by a senior pathologist without prior knowledge of the diagnosis to avoid bias. Features of the granulomas were recorded, including number, maximum diameter of the largest granuloma in each specimen, location, and the presence of absence of confluence and caseation. Other histological parameters were recorded, including biopsy site, changes of goblet cells, presence of absence of architectural alteration, cryptitis and/or crypt abscesses, dysplasia, basal plasmacytosis, discontinuous inflammation, lymphoid aggregate, lymphangiectasia, fibrosis, and neural hyperplasia. They were defined as follows:

Granuloma: localized collection of epithelioid histiocytes with or without Langhans giant cells.

Maximum diameter of the largest granuloma: measured by an ocular graticule. Diameter of the largest granuloma in each specimen was recorded. Granulomas < 200 μm in maximum diameter were categorized as small, those 200–400 μm were considered medium, and those > 400 μm as large.

Caseation: structureless necrosis with karyorrhectic debris.

Confluence of granulomas: merging of the boundaries of adjacent granulomas.

Paneth cell metaplasia: the presence of Paneth cells in the distal colon.

Crypt irregularity: crypt distortion (non-parallel crypts, variable diameter or dilated cystic crypts), crypt branching or crypt shortening in more than two crypts.

Basal plasmacytosis: plasma cells are predominantly observed between the base of the crypts and the muscularis mucosae (deep one-fifth of the lamina propria) or below the crypts, alongside or penetrating the muscularis mucosae.^13^

Discontinuous inflammation: variation in the intensity of chronic inflammation within biopsies from a site.

### Statistical Methods

The data were analyzed using SPSS 13.0 software. The χ^2^ and Fisher's exact probability tests were used to evaluate differences in the frequency of the various histological and endoscopic parameters in the ITB and CD groups. The Mann-Whitney U test was used to compare mean values of appropriate parameters in the two groups.

## RESULTS

### Clinical Features

A total of 16 patients with ITB and 52 patients with CD were studied. The mean age at diagnosis of the patients with ITB was 36 ± 13 years (range, 18–64 years), while that of patients with CD was 26 ± 9 years (range, 13–60 years) (*P* = 0.008). The male:female ratio was 2.2:1 (11:5) in patients with ITB and 1.6:1 (32:20) in patients with CD. Biopsy specimens were obtained from 39 sites of the patients with ITB and 164 sites of the patients with CD. The ileocecum was the most common site of ITB (46.1%) and CD (43.3%).

### Granuloma Features

The features of granuloma in patients with ITB and CD are shown in Table 1.

**TABLE 1:**
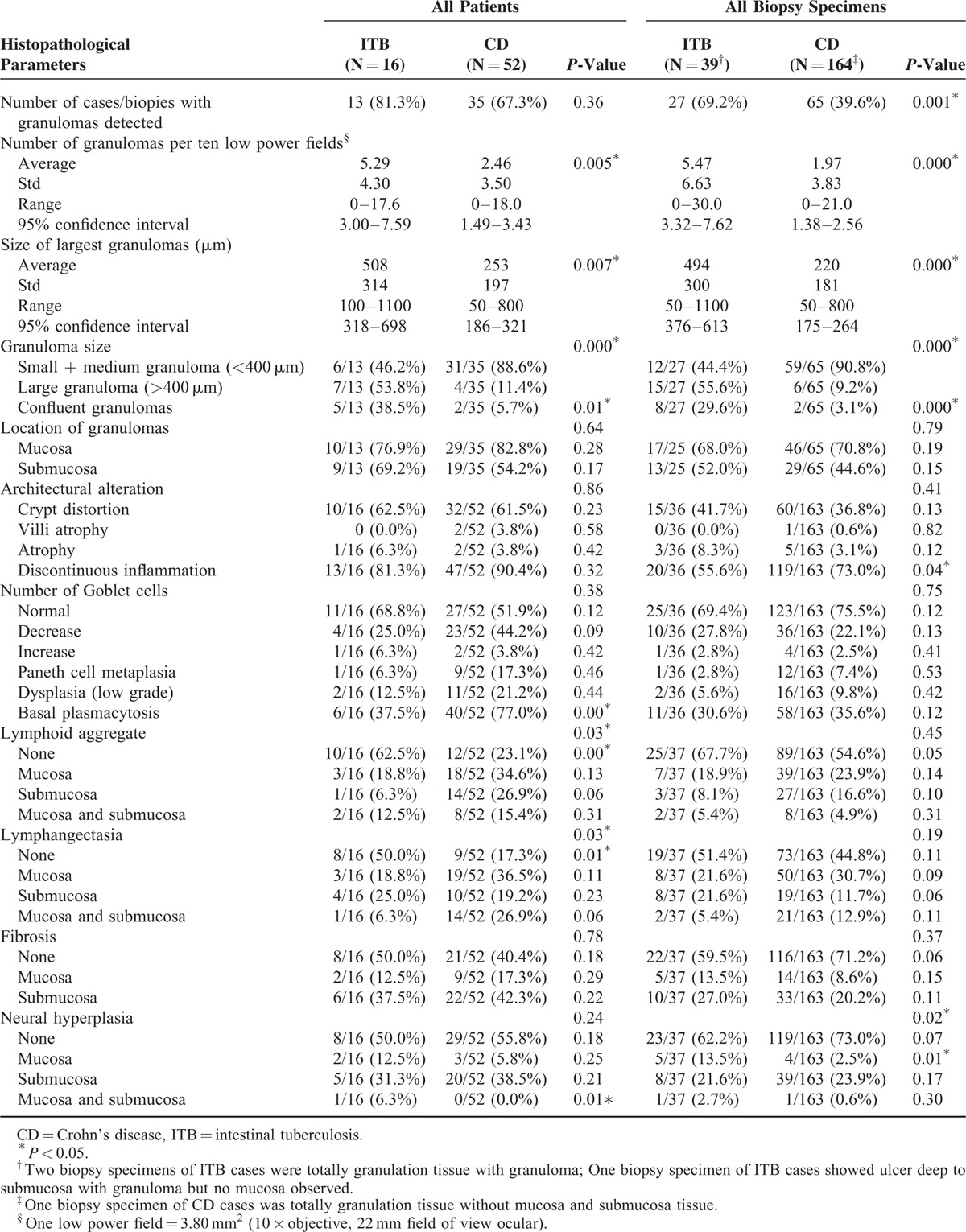
Histopathological Features in Patients With Intestinal Tuberculosis (ITB) and Crohn's Disease (CD)

Granulomas were present in 13/16 (81.3%) of ITB cases and in 35/52 (67.3%) of CD cases (*P* = 0.36) as well as in 27/39 (69.2%) of biopsy specimens of ITB cases and in 65/164 (39.6%) of CD cases (*P* = 0.001). The most common site for granulomas was the ileocecum in both ITB cases (11/27, 40.7%) and CD cases (30/65, 46.1%). As for colonic specimens, the majority of granulomas was found in the transverse colon (7/16, 43.5%) and the ascending colon (6/16, 37.5%) in the ITB cases as well as in the transverse colon (11/35, 31.4%) and the sigmoid colon (10/35, 28.5%) in the CD cases.

**FIGURE 1:**
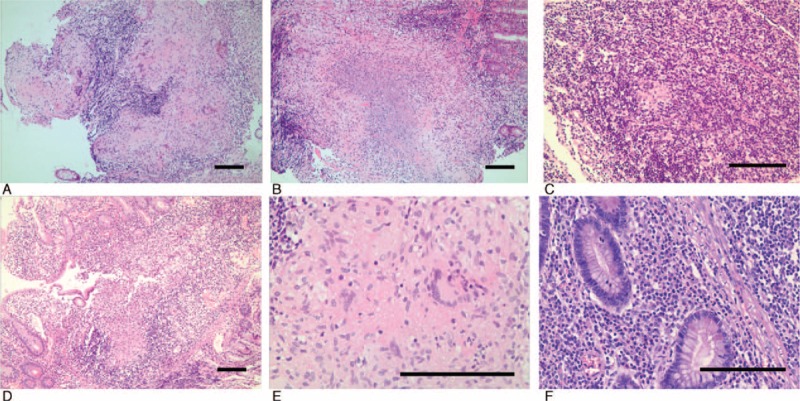
Histopathological features of intestinal tuberculosis (ITB) and Crohn's disease (CD) in endoscopic biopsy specimens. ITB is characterized by numerous large and confluent granulomas (A), while central necrosis (B) and Langhans giant cells (E) are common. CD is characterized by occasional small granulomas (C). Although large granulomas are rarely found in CD cases, they are poorly organized (D). Basal plasmacytosis was detected in CD (F) (bar = 100 μm).

Granuloma density was measured as the number of granulomas in 10 low power fields (10 × objective, 22 mm field of view ocular; each low power field = 3.80 mm^2^). An average 5.29 ± 4.30 granulomas per 10 low power fields was found in ITB cases compared with 2.46 ± 3.50 in CD cases (*P* = 0.005). In ITB cases, granulomas were larger (mean widest diameter, 508 ± 314 μm; range, 100–1100 μm) (Figs. 1A–B), and large granulomas (>400 μm in diameter) were found in 53.8% (7/13) of cases. There was one ITB case with only small granulomas (<200 μm in diameter). In contrast, CD cases had smaller granulomas (mean widest diameter, 253 ± 197 μm; range, 50–800 μm) (Fig. 1C), most of which were small and medium size granulomas (<400 μm in diameter) (31/35, 88.6%). Although large granulomas were found in four CD cases (Fig. 1D), they were immature in appearance without confluent, caseation, or Langhans giant cells. Confluent granulomas were common in ITB cases (5/13, 38.5%) (Fig. 1A) but rare in CD cases (2/35, 5.7%). Central necrosis was seen in only one ITB case (Fig. 1B) and in no CD cases. There was no typical caseation in the series. Typical Langhans giant cells were found in ITB cases (Fig. 1E) but not CD cases.

The detection rates of mucosal granulomas and submucosal granulomas in ITB cases were similar (76.9% [10/13] vs. 69.2% [9/13]). Although there were more mucosal granulomas than submucosal granulomas in the CD cases (82.2% [29/35] vs. 54.2% [19/35]), the difference in granulomas location between CD and ITB cases did not reach statistical significance.

### Other Mucosa Changes

Other mucosa changes in ITB and CD cases are shown in Table 1. Basal plasmacytosis was more frequent in CD than in ITB cases (77.0% [40/52] vs. 37.5% [6/16], *P* = 0.000) (Fig. 1F). Lymphoid aggregate and lymphangiectasia were more common in CD than in ITB cases (76.9% [40/52] vs. 37.5% [6/16], *P* = 0.000; 82.7% [43/52] vs. 50.0% [8/16], *P* = 0.010, respectively). Other features seen in CD and ITB cases were not discriminatory, including discontinuous inflammation (90.4% [47/52] vs. 81.3% [13/16], *P* = 0.32), crypt distortion (61.5% [32/52] vs. 62.0% [10/16], *P* = 0.23), Paneth cell metaplasia (17.3% [9/52] vs. 6.3% [1/16], *P* = 0.46), dysplasia (21.2% [11/52] vs. 12.5% [2/16], *P* = 0.44), fibrosis (59.6% [31/52] vs. 50% [8/16], *P* = 0.18), and neural hyperplasia (44.2% [23/52] vs. 50.0% [8/16], *P* = 0.18).

### Relationship Between Endoscopic and Histopathological Findings

**FIGURE 2:**
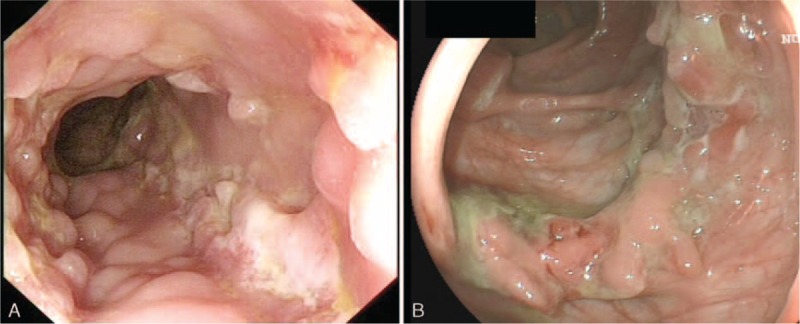
Endoscopic features of intestinal tuberculosis (ITB) and Crohn's disease (CD). Longitudinal ulcer and cobblestone appearance in CD (A). Circumferential ulcer in ITB (B).

Comparisons of endoscopic features between ITB and CD cases and relationship between endoscopic and histopathological findings are shown in Tables 2 and 3. Longitudinal ulcer (Fig. 2A), aphthous ulcer, and cobblestone appearance were only seen in CD cases (34.6% [18/52], 21.2% [11/52], and 23.1% [12/52], respectively). Circumferential ulcers (Fig. 2B) were more common in ITB cases than in CD cases (25.0% [4/16] vs. 7.7% [4/52]), but the difference did not reach statistical significance. The majority of cases with longitudinal ulcers had granulomas (16/18, 88.9%). Confluent granulomas were not found in cases with longitudinal ulcer, aphthous ulcer, or cobblestone appearance. Small and medium sized granulomas were more common in cases with irregular ulcer than in large granulomas (85.7% [30/35] vs. 14.3% [5/35], *P* = 0.02). Basal plasmacytosis was exclusively detected in cases with longitudinal ulcer and cobblestone appearance. There were no significant differences between histopathological changes and endoscopic changes in architectural alteration, changes of goblet cells, and dysplasia.

**TABLE 2:**
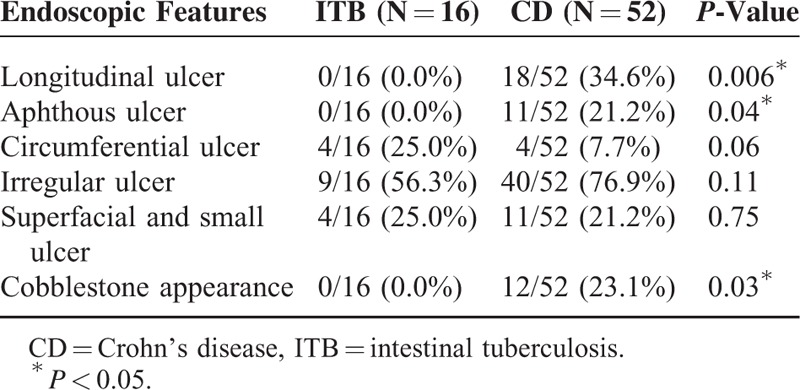
Endoscopic Features in Patients With Intestinal Tuberculosis (ITB) and Crohn's Disease (CD)

**TABLE 3:**
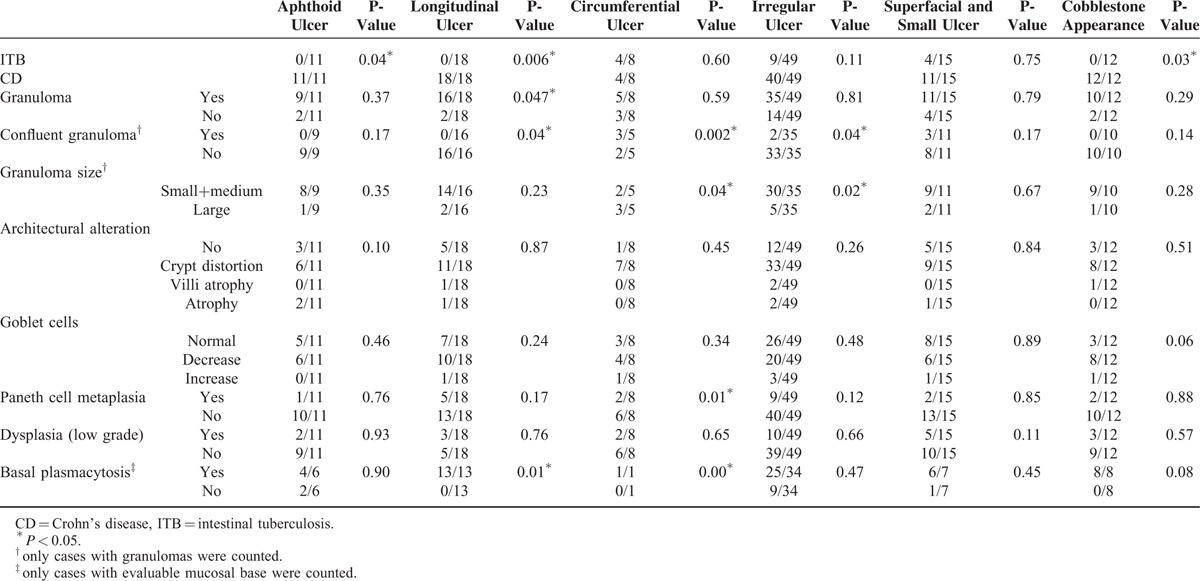
Relationship Between Endoscopic and Histopathological Findings in Patients with Intestinal Tuberculosis (ITB) and Crohn's Disease (CD)

## DISCUSSION

**FIGURE 3:**
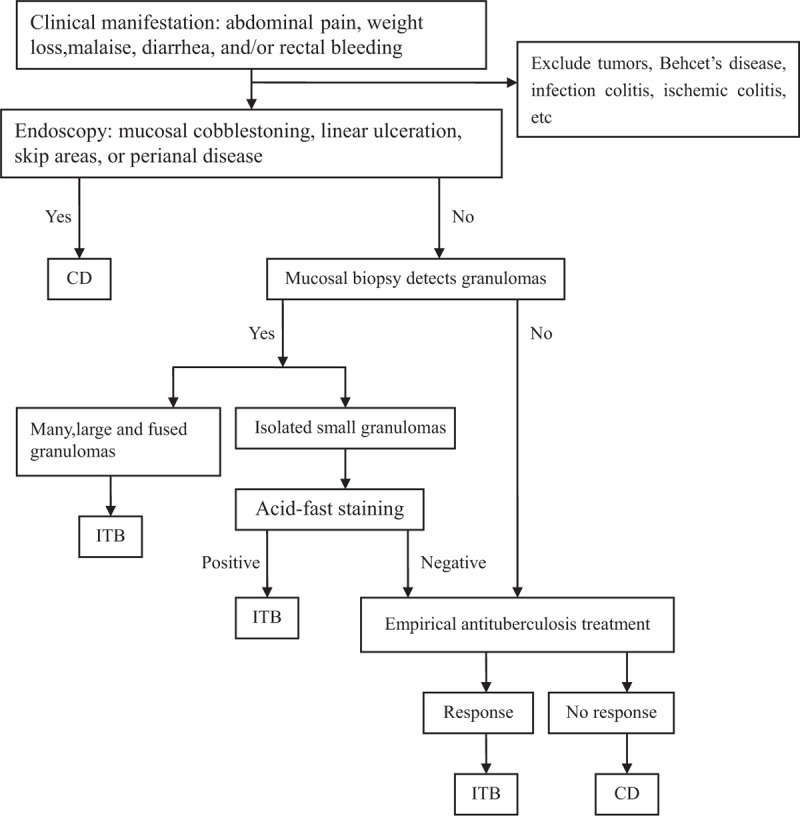
Diagnostic flowchart of differential diagnosis between intestinal tuberculosis (ITB) and Crohn's disease (CD).

Diagnoses of ITB or CD are based on a combination of clinical, endoscopic, radiological, and histological parameters, and antituberculosis treatment responses can provide clues in unclear cases (Fig. 3). Histopathological features of CD on biopsy specimens have been well characterized (eg. focal or patchy inflammation, patchy crypt irregularity, and non-caseous granulomas). However, the features of biopsy specimens on ITB have not been well described other than caseous granulomas. In the present study, we aimed to find some useful differential histopathological features that can help distinguish the two diseases by analyzing a cohort of cases with confirmed diagnoses. Here we compared the histopathological features of ITB and CD on endoscopic mucosal biopsies and explored the relationship between endoscopic lesions and histopathological findings. We found that, among all features that showed significant difference between ITB and CD, features of granulomas (eg. density, size, confluence) were the most useful distinguishing features, and the detection rate of granulomas was high in cases showing longitudinal ulcers on endoscopy. Basal plasmacytosis was exclusively detected in cases with longitudinal ulcer and cobblestone appearance.

The presence of granulomas is the characteristic histopathological feature of both ITB and CD. The granuloma detection rates in intestinal mucosal biopsies varied greatly in the literature, 50–80% in ITB cases^14–16^ and 15–65% in CD cases.^17^ ITB is characterized by numerous large, well-defined granulomas, which often feature caseation and confluence.^18,19^ CD is characterized by fewer smaller and poorly organized granulomas without confluence or caseation.^17,18^ Granulomas were denser in ITB cases than in CD cases; the density difference was believed to be one of the differentiating features between ITB and CD cases. Although the number of granulomas per biopsy fragment or biopsy site have been used as evaluation parameters of granuloma density in the literature,^20,21^ biopsy fragments size and number may vary among cases, and the data may be less comparable. In the present study, we measured granuloma density as the number of granulomas per 10 low power fields (10 × objective; 22-mm field of view ocular; one low power field = 3.80 mm^2^), which made the data more standard and comparable. The presence of five or more granulomas in one biopsies segment favors the diagnosis of ITB.^19^ Large granulomas (>400 μm in diameter) favors ITB, while small granulomas (<200 μm in diameter) favors the diagnosis of CD.^4,19,20,22^ Granuloma size on mucosal biopsy of CD is 25–350 μm.^23^

Characteristic endoscopic features of ITB and CD have been well described.^9–11^ ITB is characterized by transverse ulcers, nodularity, and hypertrophic lesions resembling masses, while CD is characterized by aphthous or longitudinal, deep, fissuring ulcers and a cobblestone appearance. A prospective study showed aphthous ulceration, linear ulceration, and superficial ulceration was more common in CD than in ITB (54% vs. 13%, 30% vs. 7%, and 51% vs. 17%, respectively). A cobblestone appearance was only seen in CD (17%). Nodularity of the colonic mucosa was significantly more common in patients with ITB than in those with CD (49% vs. 24.5%, respectively).^14^ An endoscopic scoring system has been introduced to differentiate CD and ITB. Four endoscopic parameters (anorectal lesions, longitudinal ulcers, aphthous ulcers, and cobblestone appearance) were indicative of CD, while another four parameters (involvement of fewer than four segments, a patulous ileocecal valve, transverse ulcers, and scars or pseudopolyps) were indicative of ITB. The diagnosis was considered CD when the total score for the eight parameters was greater than zero, and considered to be TB when the total score was less than zero.^24^ However, no studies have explored the relationship between endoscopic appearance and histopathological changes. The present study found that majority of cases with longitudinal ulcer detected granulomas. Confluent granulomas were not found in cases with longitudinal ulcer, aphthous ulcer, and cobblestone sign. Basal plasmacytosis was exclusively detected in cases with longitudinal ulcer and a cobblestone appearance.

Obtaining appropriate tissues for biopsy is a prerequisite for a reliable diagnosis. According to the European Crohn's and Colitis Organization and the European Society of Pathology consensus statement on the histopathological diagnosis of inflammatory bowel disease, multiple biopsies from five sites around the colon (including the rectum) and ileum should be obtained. Multiple biopsies imply a minimum of two samples from each site.^13^ Multiple biopsies are more informative than single tissues because they display a distribution pattern of inflammation and an increased granuloma detection rate.

Our study had some limitations. Although we tried to avoid bias by collecting consecutive cases diagnosed between 2010 and 2013 in our hospital and having the biopsy slide examined by a senior pathologist who was blinded to the diagnosis of each case, statistical bias may have been caused by the much smaller group of ITB cases compared to the number of CD cases. However, our hospital is one of the largest inflammatory bowel disease treatment centers in China, and it has more patients with CD than with ITB. Patients with a definite diagnosis of tuberculosis are not admitted to our hospital. As a result, we were not able to collect a large number of ITB cases. In addition, endoscopic normal mucosa samples were not taken in every case. As a result, our analytic data only included endoscopic abnormal mucosa, whereas data of normal mucosa were not analyzed. Pulimood et al^18^ found that some mucosal changes were different between ITB and CD in segments distant from those with a granulomatous response, eg. deep ulceration and moderate or severe chronic inflammation were identified in CD cases (19% and 38%, respectively) but not in ITB cases. Moreover, we were not able to match the endoscopic abnormalities and histopathological changes of biopsy tissue for every case because some sites of endoscopic abnormality in some early cases were not recorded in detail. We are now using standard endoscopy and biopsy processes, which enables us to obtain complete information about both endoscopy and histopathology for further study. The point-to-point comparison between endoscopy appearance and histopathological features may provide useful information to guide a precise biopsy for pathological diagnosis.

Although acid-fast staining and *M. tuberculosis* culture have high positive predictive values for an ITB diagnosis, they are not perfect methods. The identification of acid-fast bacilli on intestinal biopsies of ITB was 25–36% in the literature.^6,25–27^ In the present cohort, all ITB specimens were negative for acid-fast staining. *M. tuberculosis* culture may take several weeks, resulting in delayed diagnostic confirmation and treatment initiation.

Molecular techniques have been introduced to detect *M. tuberculosis* in histopathological specimens and differentiate ITB from CD. Detection of the *IS6110* gene in *M. tuberculosis* through an in-house method was widely used, with controversial sensitivity of 5.5–83%.^5,7,28^ Some new products and methods have been tried to increase PCR sensitivity and specificity. In situ PCR enables the amplification of target sequences within intact cells with a sensitivity of 30%.^29^ Real-time PCR assays exhibited a higher sensitivity of 66.7% and decreased contamination risks.^7^ PCR kits targeting the *IS6110* and *MPB64* genes (a different target detecting the *M. tuberculosis* genome) simultaneously showed a sensitivity of 45.5%.^5^ However, all methods failed to obtain high sensitivity on the endoscopic biopsy specimens, which may have been caused by paucibacilli in the small quantity of available tissue, limited number of sections used for the DNA extraction, and effect of formalin on DNA fragmentation and cross-linking.^7,29^ Moreover, it may occasionally be positive in patients with CD.^30,31^ As currently used, TB PCR on biopsy samples has a high positive predictive value but a very low negative predictive value.

Furthermore, the use of immunohistochemistry has been attempted to differentiate between ITB and CD. IHC staining for VP-M660, a monoclonal antibody targeting the 38-kDa antigen of *M. tuberculosis,* was positive in 73% of TB biopsy specimens, whereas only 7% of CD cases were stained. The specificity of VP-M660 was 93%.^32^ It showed granular cytoplasmic staining for *M. tuberculosis* in IHC staining, which is considered to be due to bacillary fragments or debris.^33^ Angiotensin converting enzyme (ACE) level has been evaluated in CD and ITB biopsy specimens by IHC staining. ACE expression was graded as an immunoreactive scoring system in which a higher degree of ACE expression indicated a higher possibility of CD.^34^

Granulomas can be observed in other infectious colitis types. Granulomas suggest *Mycobacterium* sp., *Chlamydia* sp., *Yersinia pseudotuberculosis*, and *Treponema* sp., whereas microgranulomas suggest *Salmonella* sp., *Campylobacter* sp., and *Yersinia enterocolitica*. Giant cells suggest *Chlamydia* sp.^35^ However, infectious colitis usually causes symptoms of acute abdominal pain and diarrhea, which are self-limited processes. CD and yersiniosis may be difficult to distinguish and have a long and complicated relationship. *Yersinia* pseudotuberculosis is characterized by a granulomatous process with central microabscesses, while *Yersinia enterocolitica* showed granulomas accompanied by acute inflammation and hemorrhagic necrosis. Features that may favor CD include cobblestoning of the mucosa endoscopically and chronicity microscopically, including crypt distortion, thickening of the muscularis mucosa, and prominent neural hyperplasia.^36^ Sarcoidosis is also characterized by the formation of non-caseous granulomas. However, this systemic disease rarely involves the gastrointestinal tract. Only a few cases of intestinal sarcoidosis have been reported to date. Symptoms include chronic abdominal pain, diarrhea, and weight loss. Endoscopy showed aphthous erosions, multiple nodules, polyps, obstructive lesions, stenosis, or small punctuate bleeding sites. Intestinal sarcoidosis may resemble CD when present in the colon and terminal ileum. Histopathological features that can distinguish it from CD include the presence of calcium and protein inclusions within the cytoplasm of multinucleated giant cells (Schaumann bodies) and a lack of fistulas.^37^ Moreover, cases of sarcoidosis coexisting with CD are extremely rare,^38,39^ which implies that the two diseases may share some genetic or immunologic alterations.

In conclusion, the characteristics of granulomas may be the most important feature to distinguish between CD and ITB cases. However, the histopathology of these diseases may overlap on endoscopic biopsy specimens. Thus, an accurate diagnosis should consider both clinical endoscopic features and pathological findings.
